# A 3D-Printed Modular Microreservoir for Drug Delivery

**DOI:** 10.3390/mi11070648

**Published:** 2020-06-30

**Authors:** Farzad Forouzandeh, Nuzhet N. Ahamed, Meng-Chun Hsu, Joseph P. Walton, Robert D. Frisina, David A. Borkholder

**Affiliations:** 1 Microsystems Engineering, Rochester Institute of Technology, Rochester, NY 14623, USA; ff7667@rit.edu (F.F.); nn5878@rit.edu (N.N.A.); mh4060@rit.edu (M.-C.H.); 2 Department of Chemical & Biomedical Engineering, Global Center for Hearing & Speech Research, University of South Florida, Tampa, FL 33612, USA; jwalton1@usf.edu (J.P.W.); rfrisina@usf.edu (R.D.F.); 3 Department of Communication Sciences & Disorders, Global Center for Hearing & Speech Research, University of South Florida, Tampa, FL 33612, USA; 4 Department of Medical Engineering, Global Center for Hearing & Speech Research, University of South Florida, Tampa, FL 33612, USA

**Keywords:** modular microfluidics, drug delivery, microreservoir, implantable, transdermal, 3D-printing

## Abstract

Reservoir-based drug delivery microsystems have enabled novel and effective drug delivery concepts in recent decades. These systems typically comprise integrated storing and pumping components. Here we present a stand-alone, modular, thin, scalable, and refillable microreservoir platform as a storing component of these microsystems for implantable and transdermal drug delivery. Three microreservoir capacities (1, 10, and 100 µL) were fabricated with 3 mm overall thickness using stereolithography 3D-printing technology, enabling the fabrication of the device structure comprising a storing area and a refill port. A thin, preformed dome-shaped storing membrane was created by the deposition of parylene-C over a polyethylene glycol sacrificial layer, creating a force-free membrane that causes zero forward flow and insignificant backward flow (2% of total volume) due to membrane force. A septum pre-compression concept was introduced that enabled the realization of a 1-mm-thick septa capable of ~65000 leak-free refill punctures under 100 kPa backpressure. The force-free storing membrane enables using normally-open micropumps for drug delivery, and potentially improves the efficiency and precision of normally-closed micropumps. The ultra-thin septum reduces the thickness of refillable drug delivery devices, and is capable of thousands of leak-free refills. This modular and scalable device can be used for drug delivery in different laboratory animals and humans, as a sampling device, and for lab-on-a-chip and point-of-care diagnostics applications.

## 1. Introduction

Controlled drug delivery systems have drawn interest in recent decades for enhancing therapeutic interventions due to their ability to provide more consistent dosing over time and improving patient comfort, safety, and compliance. Microscale reservoir-based systems have been utilized in systemic administration and also site-directed delivery to limit systemic exposure side effects [1]. The miniaturized size of these systems enables site-directed delivery to relatively inaccessible sites or specific tissues in small rodents and humans. Further, to reduce the biological and mechanical impact to the target tissues, the pumping mechanisms in these devices often provide precise control over the flow rate, especially in low ranges (0.01–1 µL/min).

Reservoir-based drug delivery devices with capacities ranging from a few to hundreds of microliters have been widely used in implantable and transdermal drug delivery formats. Implantable drug delivery systems enable some of the most innovative and elegant drug delivery concepts to address current medical needs [1]. These devices typically include a reservoir for drug storage, a microtubing, and a micropump to propel the drug from the reservoir to the target organ through the microtubing. Implantable drug delivery systems with reservoir capacities of 1.2, 15, 20, 60, and 130 µL were used for the delivery of edaravone and unoprostone isopropyl to the posterior segment of the eye [2], the rapid vasopressin release for treating hemorrhagic shock [3] and ambulatory emergency care [4], the delivery of ranibizumab into the vitreous cavity [5], and the infusion of fluorescein isothiocyanate-dextran-labeled dextran marker into perilymph of guinea pigs for inner ear drug delivery [6], respectively. Transdermal administrations have been used widely because of their ease of use, minimal invasiveness, capability for self-administration, and low sustainable cost [7]. These devices generally consist of a reservoir, a microneedle, and a pumping mechanism for effective delivery through the microneedles [8,9]. Transdermal drug delivery devices with reservoir capacities of 5, 12, 20, 70, and 100 µL were used for trace blood tests [10], insulin administration to diabetic rats [11], general pharmaceutical applications [12], fast-onset and sustained delivery of lidocaine [13], and general transdermal drug delivery applications [8].

The drug reservoir is an under-studied, under-developed component of drug delivery systems. Some drug delivery systems are designed to be refillable, especially microscale drug delivery systems, where refill ports assist in reducing the overall size by eliminating the need for a large reservoir [7]. Refills occur by injections via sharp, thin, non-coring needles into a septum located in a designated refill port in the system housing [14,15]. The septa are usually made of silicone rubber [14] with self-healing capabilities [16], due to their high resilience [17], high deformability, and self-adherence [18,19]. Refill ports typically have a baseplate for penetration depth limitation and a cavity between the septum and baseplate with an exit channel to provide a fluidic interconnection to a storing area [20,21,22]. In some cases, a raised ridge [22,23] or a ring with a different color [24] is incorporated into the refill port to assist palpation, localization, or visual identification for users. Although there are several microsystems in the literature or on the market that use septa for refilling, only a few of them report the number of punctures they can withstand without leakage. For instance, Lo et al. developed a microdevice for the treatment of ocular diseases with a polydimethylsiloxane (PDMS) membrane refill port capable of 24 punctures under ~5 and ~30 kPa backpressures for membrane thicknesses of 250 µm and 673 µm, respectively [24,25,26,27]. PROMETRA^®^ programmable pumps (InSet Technologies Incorporation, NJ, USA) use silicone rubber for the septa, rated to withstand an average of 1000 punctures. Hamilton^®^ GC Septa are rated for a maximum of 100 injections with 26s Ga needles, with a minimum thickness and diameter of 3 and 5 mm.

Ideally, the reservoir as a storing unit should not affect pumping performance. Usually, the reservoirs are made of elastomeric membranes without pre-compression integrated into pumping mechanisms. As the drug is extracted from the reservoir, the membrane deforms, resulting in an induced stress that tends to restore the membrane to its original shape; a restoring force. This force results in a non-negligible backward flow after pumping has stopped, which is typically counteracted by deploying either a normally-closed fluidic channel or check valves [24,28,29]. The restoring force of the reservoir membrane prevents it from being integrated to normally-open pumps as it dramatically affects the pumping efficiency. Even when integrated into normally-closed pumps, the restoring force still reduces pumping efficiency and accuracy due to its variable negative pressure, which needs to be considered in the design. On the other hand, the reservoir membranes in some designs are pre-compressed to serve as a pumping mechanism [9,30]. For instance, the ithetis pump (ithetis pump; Antlia SA, Lausanne, Switzerland) uses an expandable pouch-reservoir that pressurizes the fluid in the reservoir, with a valve enabling flow. However, in this design concept the membrane force changes as the reservoir discharges, necessitating further design considerations to avoid potential inconsistencies and inaccuracies in pumping. To the best of our knowledge, no microsystems have been reported that utilize a reservoir membrane with negligible forward or backward flow due to membrane force for the integration into micropumps.

Laboratory animal models play an essential role in understanding and treating human diseases. The most common animal models in research are rats and mice [31], with mice offering the advantage of genetic manipulation [32]. Therapy development in these animal models requires system miniaturization, with scalability preferred to enable clinical translation to humans. System thinness is a crucial factor for these animal drug delivery systems but is also important in size-constrained implantable and transdermal drug delivery in humans. Three-dimensional printing technology enables the fabrication of thin, miniature, and scalable devices with biocompatible materials, and has emerged as a powerful tool in the creation of miniaturized drug delivery systems.

To the best of our knowledge, no stand-alone microreservoir has been reported that takes into account the aforementioned considerations for transdermal and implantable drug delivery microsystems. This necessitates a modular microreservoir as an independent component for the integration to various pumping mechanisms without impacting pumping performance, enabling precise drug delivery for transdermal and implantable applications. Here we present a stand-alone, miniature, thin, scalable, and refillable microreservoir platform to be integrated into pumping mechanisms as a storing component for implantable and transdermal drug delivery microsystems. This microreservoir platform has a small footprint and a planar form factor for subcutaneously implantable and transdermal drug delivery for small animal models, while being readily scalable for larger animals and human translation. Using 3D-printing technology to fabricate the structure, microreservoirs with three capacities of 1, 10, and 100 µL were demonstrated and characterized. The microreservoir membrane was made with a thin parylene-C layer deposited over a polyethylene glycol (PEG) sacrificial layer, providing a class VI biocompatible environment for drug storage that induces a minimal force on the stored drug. A novel pre-compression technique is introduced for the fabrication of ultra-thin septa. Benchtop characterization experiments were performed on the membrane to evaluate the flow due to membrane force, total extraction percentage, and the number of performance cycles. Further, *in vitro* experiments were performed to evaluate the device performance for implantable and transdermal applications, where drug models with different viscosities were used as the working fluid. In addition, the ultra-thin septum was tested to determine the number of leak-free punctures it could endure against high backpressures.

## 2. Materials and Methods

### 2.1. System Operation Concept and Architecture

**Figure 1:**
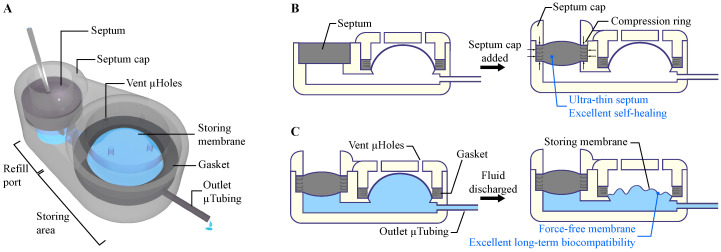
(**A**) Conceptual schematic of the microreservoir. The structure of the microreservoir is made of two main sections: the refill port for refilling the microreservoir by injections through a septum, and the storing area for drug storage. The structure is made with 3D-printing technology using a biocompatible resin, enabling printing micro-scale features that are readily scalable, allowing applications from mice to humans. (**B**) The septum cap incorporates a compression ring that causes the septum to be compressed both vertically and laterally. The vertical compression ensures sealing from the bottom and holds the septum during numerous injections. The lateral compression induces internal stress in the septum to improve its self-healing capability. This feature enables a significant reduction of the septum thickness while maintaining high resistance to leakage due to punctures. (**C**) The storing membrane is made of a pre-formed, dome-shaped, thin parylene-C layer, enabling zero forward flow and negligible backward flow after partial/full discharge of the microreservoir. When integrated to a pumping mechanism, this feature decouples the storing and pumping roles, which allows precise control over the flow rate. Finally, since all the components and coatings of internal and external surfaces are class VI biocompatible, long-term drug storage and minimal inflammation and infection for tissues in contact with the device are ensured.

To build a microscale and scalable structure, the stereolithography (SLA) fabrication approach was employed. The Formlabs SLA 3D-printer (Form 2, Formlabs Inc., Somerville, MA, USA) was used to fabricate a rigid biocompatible structure comprising a refill port, storing area, an outlet port, microchannels to connect them, and a baseplate. This rigid baseplate controls the penetration depth of the refill needle while providing a surface to form the reservoir membrane. The rigid frame protects the enclosure and ensures sufficient rigidity for handling. Figure 1A shows a schematic view of the overall structure of the microreservoir.

The refill port has a 2.5 mm opening for the septum and a built-in septum stopper to ensure space between the septum and the baseplate, allowing the fluid flow into the storing area. A 2.5-mm septum is micro-molded in a cylindrical shape with silicone rubber, followed by parylene-C deposition. A cap with a raised ring (compression ring) was placed and fixed on the septum to provide vertical compression, which improves sealing and facilitates fixation of the septum in the port during injection, a reported challenge in existing literature [33,34]. The compression ring also causes the septum to laterally push the port wall, inducing a lateral compression in the septum, and enhancing its self-healing properties. This lateral compression makes it possible to reduce the septum thickness to only 1 mm with high resistance to leaking after refill injection punctures. A raised ridge is designed on top of the septum cap to enable subcutaneous palpation and visual access for refilling. Figure 1B depicts the pre-compression concept of the refill septum.

To provide an inert and biocompatible environment for therapeutic compounds, the stored compound was designed to be encapsulated with parylene-C, a USP Class VI material suitable for implantable and transdermal devices [35]. Furthermore, parylene-C has less permeability to liquid compared to other conventional materials used in the literature for reservoir membranes, such as PDMS [25] and MDX-4-4210 [28]. After the first layer of parylene-C deposition, molten PEG was deposited on the storing area and solidified to build a biocompatible sacrificial layer, a technique that has been used to form parylene-made cavities [28]. A second parylene-C layer was deposited on the PEG sacrificial layer to build the thin pre-formed dome-shape storing membrane. The seal between two parylene-C layers was enhanced using a compressed biocompatible silicone gasket on the edge of the storing area. The thicknesses of the membranes for three different capacities (1, 10, and 100 µL) were optimized to withstand 100 kPa backpressure, which is four times larger than the maximum physiological backpressure in humans [36]. This backpressure was also used as a criterion for septum characterizations. For all capacities, the overall thickness of the device was set to 3 mm, to be suitable for long-term subcutaneous implantation and transdermal applications.

The pre-formed shape and thinness of the storing membrane make it force-free, enabling zero forward flow and negligible backward flow due to the membrane force on the stored drug. (See Figure 1C). The force-free characteristic of the storing membrane facilitates precise control of the flow rate for integrated pumping mechanisms, by decoupling storing and pumping functions. It also eliminates the necessity of a check valve in the integrated micropump, enabling the use of normally-open micropumps in drug delivery applications with low/zero backpressures. Furthermore, when integrated into normally-closed micropumps, the permanent negative pressure on the check valves would be eliminated, yielding a potentially higher pumping efficiency. The use of biocompatible materials for the fabrication of the structure, storing membrane, sacrificial materials, septum, gasket, and for coating all internal and external surfaces, ensures a long-term storage capability of the device and minimal inflammation or infection for tissues in direct contact with the device.

### 2.2. Fabrication Process

The microreservoir substrate was 3D-printed (Form 2, Formlabs Inc., Somerville, MA, USA) with a rigid biocompatible [37] material (Dental SG resin, Formlabs Inc., Somerville, MA, USA). The design included a hole of 2.5-mm diameter and 1-mm depth for the septum, storing area, outlet port, fluidic interconnections between them, and a baseplate. In the storing area, a raised circle-shaped ring was created for placing the gasket (gasket ring), with an angled wall to the baseplate to reduce dead volume. In the refill port, a septum stopper with a diameter of 1.8 mm was designed to provide a seat for the septum. A biocompatible [38] polyurethane-based catheter microtubing (ID = 125 μm, OD = 250 μm; Micro-Renathane^®^ Catheter Tubing, Braintree Scientific Inc., Braintree, MA, USA) was fixed and sealed to the output port, using cyanoacrylate, which has been widely utilized to fabricate medical devices [39]. The substrate was coated with a 1-μm-thick layer of parylene-C using a PDS 2010 LABCOATER™ 2 (Specialty Coating Systems, Indianapolis, IN, USA).

Microreservoirs with three different capacities (1, 10, and 100 µL) were fabricated, where the diameters of the storing area were 1.3, 3.1, and 10 mm, respectively, considering a 3-mm overall thickness. To build the storing membrane with parylene-C, a sacrificial layer was deployed to define the capacity of the microreservoir after release. PEG (1500 Mn, melting point: 37 °C, Sigma-Aldrich, St. Louis, MO, USA) was chosen for the sacrificial layer due to its biocompatibility [40] and solubility in water. PEG was melted at 70 °C on a hotplate and mixed with a food dye (McCormick Food Color and Egg Dye, McCormick & CO., Baltimore, MD, USA) at 20:1 ratio, for visual confirmation of its release. Using a heated micropipette (70 °C), molten PEG was deposited on the storing area. To prevent PEG from flowing on the gasket ring, it was treated with a hydrophobic spray (Scotchgard™ Fabric & Upholstery Protector, 3M Co, Saint Paul, MN, USA) using a foam swab. The substrate was kept at room temperature during PEG deposition because it was empirically found that it provides a sufficiently fast cooling rate to avoid the flow of the PEG in the channel regions, resulting in a smooth PEG surface. The full volume of required PEG was deposited with a single micropipette injection.

To make the storing membrane on top of the PEG sacrificial layer, the required thickness of the second parylene-C layer was estimated based on thin-walled spherical pressure:

(1) σ=pr/2t

where *σ, p, r*, and *t* represent tensile stress on the membrane, internal pressure, storing area radius, and membrane thickness, respectively. The membrane was designed to withstand 100 kPa backpressure (*p* = 100 kPa), while the radius was designed based on overall thickness and the capacity of the device. The thickness of the parylene-C membrane for each capacity was calculated to achieve a membrane stress that is five times smaller than the tensile strength of parylene-C (69 MPa [41]). The storing membrane thicknesses were calculated at 2.7, 5.6, and 18.1 µm for 1, 10, and 100 µL capacities, respectively.

The gaskets were fabricated by micro-molding a long-term implantable biocompatible [42] silicone rubber (MED-6215, NuSil™ Technology LLC, Carpinteria, CA, USA, 1:10 ratio) in machined aluminum molds, followed by curing at 150 °C for 15 min. Gaskets are 0.5 × 0.5 mm^2^ (width and depth) with inner diameters of 1.3, 3.1, and 10 mm for capacities of 1, 10, and 100 µL, respectively. The gasket was placed on the gasket ring and fixed and compressed with a 3D-printed, parylene-C-coated storing cap (Form 2, Formlabs Inc., Somerville, MA, USA). The storing cap also covers the storing membrane to protect it from external mechanical stresses. The storing cap included vent micro-holes of 0.2 mm diameter to allow ingress and egress of air or liquids from the space between the cap and the storing membrane during filling and discharging. These holes are smaller than the smallest needle size for refilling the microreservoir (30 Ga, 311 µm OD) to avoid inadvertent punctures of the membrane during subcutaneously refilling.

**Figure 2:**
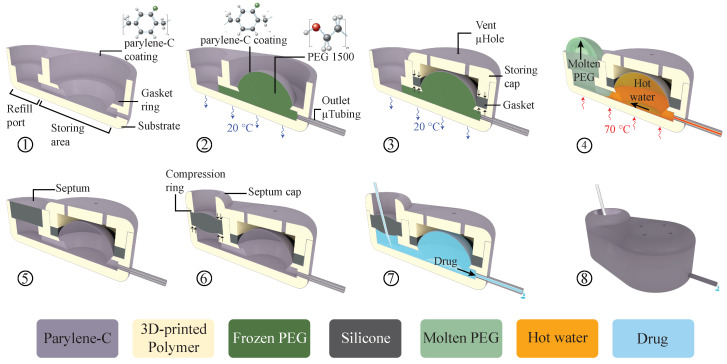
A cut-view schematic of the microreservoir fabrication process. (1) The substrate of the microreservoir was 3D-printed using a biocompatible resin, followed by 1 µm parylene-C deposition. (2) A microtubing (250 μm OD, 125 μm ID) was inserted into the substrate outlet port and sealed in place with cyanoacrylate. The substrate was held at room temperature, and molten polyethylene glycol (PEG) at 70 °C was deposited on the storing area to solidify and create the microreservoir volume. This was followed by another parylene-C deposition to form the storing membrane capable of withstanding 100 kPa backpressure. (3) A silicone gasket was fabricated using a micro-molding technique and was placed on the gasket ring. A 3D-printed parylene-C-coated cap (storing cap) was affixed on top of the storing area with cyanoacrylate, compressing the O-ring to reinforce sealing between the two parylene-C layers, and to protect the storing membrane from mechanical stress. The storing cap has vent microholes to allow egress/ingress of air or fluid between the membrane and the cap during filling/discharging. (4) The substrate was placed on a hotplate at 70 °C to melt the PEG and wash it by gentle injection of 100 mL of deionized water at 70 °C (5) A 2.5-mm diameter, 1-mm-thick septum made of long-term implantable silicone rubber was micro-molded and coated with parylene-C. The septum was placed in the refill port (2.5 mm diameter). (6) A 3D-printed, parylene-C-coated septum cap with a raised compression ring (2.5 mm OD, 1.8 mm ID) was affixed on top of the septum with cyanoacrylate to compress the septum improving sealing and self-healing. (7) Schematic cut view of the fabricated microreservoir being filled by a refill needle. (8) A schematic full-body view of the microreservoir.

Under visual observation using a microscope (Motic SMZ-168), the substrate was heated on a hotplate at 70 °C to melt the PEG, allowing it to be rinsed away with 100 mL 70 °C deionized (DI) water. A silicone (MED-6215, NuSil™ Technology LLC, Carpinteria, CA, USA, 1:10 ratio) septum with 2.5 mm diameter and 1 mm thickness was micro-molded with 3D-printed and parylene-C-coated molds and cured at 150 °C for 15 min, followed by a 1 µm parylene-C deposition. The septum was placed in the refill port, followed by placing and affixing a 3D-printed and parylene-C-coated septum cap using cyanoacrylate. The septum cap incorporated a 0.2-mm-thick compression ring (1.8 mm ID, 2.5 mm OD) to compress the septum to provide robust sealing and improved self-healing. A raised ridge (1.8 mm ID, 3 mm OD, 0.5 mm thickness) was integrated on top of the septum cap to facilitate palpation for subcutaneous refills. All surfaces were filleted to minimize potential mechanical inflammation after implantation. The full fabrication process is illustrated in Figure 2. Detailed dimensions of the components are provided in Figure S1.

### 2.3. Experimental Methods

To assess the functionality of the septum and the storing membrane, these two parts were decoupled and tested separately. The samples were fabricated following the processes described in Section 2.2. To evaluate the microreservoir for implantable and transdermal drug delivery, *in vitro* experiments with the fully integrated structure were performed on 10 µL microreservoirs.

#### 2.3.1. Septum Test

**Figure 3:**
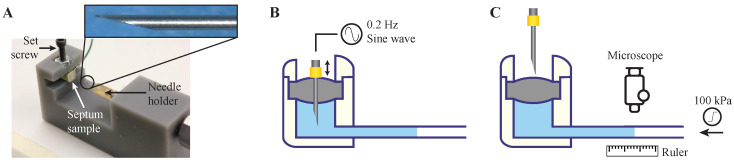
Benchtop test setup for septum characterization. (**A**) A puncture device was made to hold septum samples in a groove with a set screw and punctures were made with a needle through the septum with an automated pneumatic mechanism. Inset: a 30 Ga non-coring 12° beveled needle used for puncture test. (**B**) The septa test samples were punctured in a single point with the puncture device fed by a 0.2 Hz sine wave signal. (**C**) After a set number of punctures, the pneumatic system was stopped, and backpressure was gradually increased to 100 kPa. At the same time, the fluid front displacement was quantified by a microscope with a resolution of 2.4 nL/min.

The septum was tested utilizing separately fabricated septum samples, consisting of a 3D-printed refill port with septum stopper, coupled to a Tygon tubing outlet (0.508 mm ID, 1.52 mm OD). A pneumatic puncture device was designed and fabricated to hold the septum sample and automatically puncture through the septum in a single location, to test the worst-case scenario [24,33]. The structure of the pneumatic puncture device was 3D-printed using Grey Pro resin (Formlabs Inc., Somerville, MA, USA), with a groove for the placement of the test sample, which was fixed with a set screw. A small cylinder (SM-3-1, Clippard Co., Cincinnati, OH, USA) on top of the sample was connected to an electronic pressure regulator (ITV2030-31N2BL4, SMC Co., Tokyo, Japan). The pressure regulator was fed with sine waves of 0.2 Hz by a signal generator to simulate a realistic manual puncture speed. The needle tip was affixed to a 3D-printed needle holder, which was press-fit to the piston of the cylinder. Figure 3 shows the pneumatic puncture device with a 30 Ga needle.

Commercially available 27 and 30 Ga (413 and 311 μm OD) needles were used for the puncture tests. These sizes were selected as a trade-off between maximizing needle and septum lifetime: the needles need to be large enough to puncture the skin and the septum without bending the tip, while small enough to minimize damage to the septum. In the literature, 30 Ga needles were used for septum characterization [24], and since the opening diameter of the septum cap is 1.8 mm, needle sizes larger than 27 Ga were impractical. The needles were machined into a non-coring shape to minimize damage to the septum structure [24,25,26]. The needle tip was beveled to a 12° angle, as recommended for animal injections [43].

Septum samples were prepared for puncture experiments with 27 and 30 Ga needles. To investigate the effect of the compression ring on the self-healing characteristics of the septum, one batch of samples (N = 4) was fabricated without septum caps. Instead, they were fixed and sealed in the refill port using cyanoacrylate, causing no septum pre-compression. In this experiment, 30 Ga needles were used, and the results were compared with the samples with the septum cap incorporating a compression ring punctured with 27, and 30 Ga needles (N = 4 for each).

To test the septum function, the space beneath the septum and 5 cm of the outlet tubing were filled with dyed DI water using sharp (12°) non-coring needles, and the septum samples were fixed in the pneumatic puncture device. The outlet tubing was placed on a ruler to enable quantitative observation of the fluid motion for the leakage test. A signal generator provided a 0.2 Hz sinusoidal drive to the pneumatic actuator to repeatedly puncture the septum. The number of repetitive punctures was increased logarithmically (number of injections = 10^0.2n^; *n* = 0, 1, 2, …) with the leakage test between each step. This test involved a gradual application of 100 kPa pneumatic backpressure at the outlet tubing for 1 min, while the fluid displacements in the tubing were observed and recorded with a digital microscope (USB-MICRO-250X, Plugable^®^, Redmond, WA, USA). The images were analyzed with NIH-ImageJ, resulting in a resolution of 2.4 nL/min for the leakage test. If no leakage was observed, the test continued by switching on the signal generator until the next logarithmic step. The number of punctures before leakage was found for each case based on the last value with no leakage.

#### 2.3.2. Storing Membrane Test

Microreservoir actual capacities, the forward and backward flow due to membrane force, total fluid extraction percentage, and the number of discharge/refill cycles were characterized. The storing membrane test samples were fabricated and tested separately, with a slight modification to the fabrication process explained in Section 2.2. The microreservoir inlet was directly connected to an inlet tubing, a three-way stopcock, and syringes for filling, with an outlet tubing aligned over a ruler to quantify fluid movement due to the pneumatic pressure applied on top of the storing membrane.

To fill the cavity, the pneumatic pressure was set to zero, and a syringe was connected to the inlet port and pulled to remove air from the storing area and pull the membrane down to its minimum volume. The syringe was replaced with a three-way stopcock connecting to the inlet tubing. Using a 1 mL syringe, dyed DI water was injected to fill the inlet tube up to the entrance of the storing area via visual observation using a microscope. The 1 mL syringe was replaced by a smaller syringe to accurately quantify the injected volume: a 25 µL syringe (1702 LT SYR, Hamilton Co., Reno, NV, USA) for the 1 and 10 µL capacities, and a 250 µL syringe (1725 TLL, Hamilton Co., Reno, NV, USA) for the 100 µL capacity. With the outlet tubing open, the syringe was discharged until the fluid was observed to reach the microreservoir exit. The outlet tubing was then clamped closed, followed by the discharge of the syringe to fill the storing area confirmed by visual observation using a microscope. The three-way stopcock was switched to block flow on the inlet side, while the outlet tubing clamp released to quantify the fluid volume in the outlet tubing. The actual capacity of the microreservoirs was calculated by subtraction of the fluid volume in the outlet tubing (based on fluid front displacement) from the injected fluid volume from the syringe.

**Figure 4:**
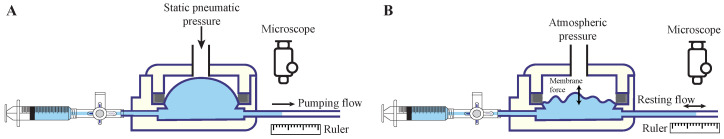
Benchtop test setup for the storing membrane characterization. (**A**) The microreservoir was filled, and the inlet was blocked using the stopcock. The fluid in the storing membrane was pumped via a static pneumatic pressure on the membrane until 20% of the capacity was discharged. (**B**) The pneumatic pressure was released, and the flow in the tubing due to membrane force was quantified under a microscope. Different tubing sizes were used for the three capacities to maintain a minimum resolution of 0.1% of the full capacity. The experiment was repeated for the next 20% of the overall capacity. The total discharged fluid after the fifth step indicates the total extraction percentage.

The forward and backward flows due to the membrane force were characterized for extracted volumes of ~20%, 40%, 60%, 80%, and 100% of the overall capacity. Pneumatic pressure was applied above the storing membrane for 1 min to induce forward fluid movement, as shown in Figure 4A. After discharge of ~20% of the volume, the pneumatic pressure was released, and the fluid front displacement in the outlet tubing was observed under a microscope for 4 min (Figure 4B). For the three capacities, three different outlet tubing sizes were used to provide a measurement resolution of 0.1% of the full capacity using a 0.5-mm graded ruler. The backward flow of the fluid front was recorded, and the experiment was repeated for the next 20% of the overall capacity. The total fluid in the outlet tubing after the fifth step indicated the total extraction percentage. Three samples for each of the three capacities were tested, with three replicates for each (N = 9). To find the number of discharge/refill cycles, four microreservoirs with 10 µL capacities along with the septa were fabricated and filled following the aforementioned procedures. The outlet microtubing was connected to a syringe pump (SP210iw, World Precision Instruments, Sarasota, FL, USA) and was programmed to discharge/refill the microreservoir from the outlet end, with an 18-s cycle time. The storing membrane was observed under a microscope to detect potential leakage.

#### 2.3.3. *In Vitro* Test

To evaluate the performance of the microreservoirs for implantable and transdermal drug delivery, 10 µL microreservoirs (with integrated septum and storing area) were fabricated following the processes described in Section 2.2, and subsequently tested. To simulate a subcutaneous implant environment, the microreservoir was submerged in normal saline at 37 °C [44]. To simulate the transdermal drug delivery environment, the microreservoirs were placed on a 37 °C [45] surface in air.

Drug solutions vary in viscosity as a function of base solution and drug concentration. Conventional injectable drugs have viscosities ranging from 1 to 20 cP [46]. Therefore, a protocol for the preparation of glycerol solutions with various viscosities was developed and the microreservoirs were tested using these drug models for both implantable and transdermal applications. To make a thin solution with a viscosity close to that of DI water, a food dye (McCormick Food Color and Egg Dye, McCormick & CO., Baltimore, MD, USA) and DI water mixture (2%, *w/w*) was prepared. To provide thicker solutions, glycerin (Glycerin 99%, PTI Process Chemicals Inc., Ringwood, IL, USA) was dissolved in the food dye in two different concentrations of 62% and 84% (*w/w*). The viscosities of the three solutions were measured with a microVISC viscometer (RheoSense, San Ramon, CA, USA), resulting in viscosities of 1.1, 19.8, and 151.7 cP covering a broad range of drug viscosities up to ~7.5 times greater than thickest conventional injectable drugs.

**Figure 5:**
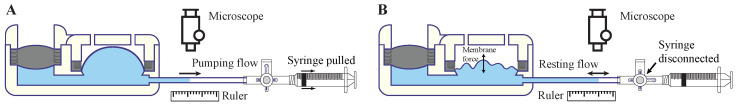
*In vitro* characterization of the microreservoirs was performed on 10 µL microreservoirs (septum and storing area integrated) to evaluate performance for implantable and transdermal applications in simulated benchtop environments. (**A**) The storing area was filled through the septum. The fluid in the storing membrane was pumped via a vacuum created by pulling an empty syringe until 20% of the capacity was discharged. (**B**) The pumping vacuum was released, and the flow in the tubing due to membrane force was quantified under a microscope. The experiment was repeated for the next 20% of the overall capacity.

The solutions were injected through the septum using 30 Ga non-coring sharp needles, and the storing area was filled following the processes described in Section 2.3.2. The forward and backward flows due to the membrane force were characterized following the procedures described in Section 2.3.2. An outlet microtubing with ID = 0.508 mm was used to provide a measurement resolution of 0.1% of the full capacity when aligned over a 0.5-mm graded ruler. The outlet was connected to an empty 1 mL syringe and pulled to create a vacuum and induce a forward flow (Figure 5A). This method of pumping was chosen instead of the pneumatic pumping, which was used in Section 2.3.2, to expose the storing membrane to the surrounding fluid to replicate *in situ* applications. After discharge of ~20% of the volume, the three-way stopcock was switched to stop discharging and expose the fluid to atmospheric pressure. The fluid front displacement in the outlet tubing was observed under a microscope for 4 min (Figure 5B). Three samples were used for each of the implantable and transdermal *in vitro* experiments using different working fluid viscosities (N = 3).

## 3. Results

**Figure 6:**
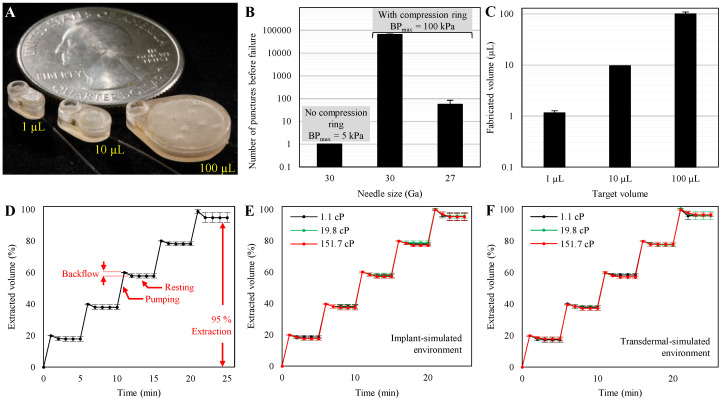
(**A**) Completed microreservoirs with capacities of 1, 10, 100 µL, showing the scalability of the design and fabrication process. (**B**) Septum characterization results showed that the samples without compression rings leaked at less than 5 kPa applied backpressure with just one puncture with a 30 Ga needle. Adding a compression ring to the septum cap increased the number of punctures before failure to ~65,000 at 100 kPa backpressure when puncturing with a 30 Ga needle. This design showed no leakage up to 55 injections at 100 kPa backpressure when punctured by a 27 Ga needle. N = 4, mean ± SD. (**C**) Fabricated volumes were 1.15 ± 0.12, 9.63 ± 0.12, and 100.04 ± 9.43 for target volumes of 1, 10, and 100 µL. N = 4, mean ± SD. (**D**) The average results of the backward flow due to the restoring force for capacities of 1, 10, and 100 µL normalized by the total volume of each. The results showed zero forward flow and insignificant backward flow (2.02% average), which occurred quickly (<2 min) after pneumatic pressure release, suggesting long-term stable behavior of the storing membrane. The last step of the experiment shows the total extraction percentage, which was 95% of the capacity. N = 9, mean ± SD. (**E**) The results of *in vitro* experiments for implantable drug delivery on fully integrated 10 µL microreservoirs with a drug model of 1.1 cP showed an average backflow of 1.69%, indicating that the microreservoir can fully operate in a subcutaneous implant environment. Further, using drug models with viscosities of 19.8 and 151.7 cP showed 1.98% and 2.59% average backflow, demonstrating that the microreservoir can work regardless of the drug viscosities up to ~7.5 times greater than those of typical injectable drugs for implantable drug delivery. N = 3, mean ± SD. (**F**) Results of *in vitro* experiments for transdermal drug delivery on fully integrated 10 µL microreservoirs with a drug model of 1.1 cP show an average backflow of 2.06%, indicating the suitability of the device for transdermal drug delivery. The results of drug models with viscosities of 19.8 and 151.7 cP showed 2.24% and 2.33% average backflow. This shows proper functionality of the device independent of drug viscosity for transdermal drug delivery. N = 3, mean ± SD.

Fabricated microreservoirs are shown in Figure 6A in comparison to a US quarter coin. The overall footprints of the microreservoirs were 5.8 × 3 (1 µL), 7.6 × 4.6 (10 µL), and 14.5 × 11.5 mm^2^ (100 µL) with a thickness of 3 mm for all. Benchtop experiments were performed to optimize the septum design for thousands of refilling injections without leakage and quantify the backward and forward flow due to membrane force. Further, the total capacity of the microreservoirs and total extraction percentages of each were assessed for all three prototype capacities. The 10-µL prototypes were evaluated for the discharge/refill test. Finally, the 10 µL microreservoirs were tested for *in vitro* application for both implantable and transdermal drug delivery by simulating the environments and different drug viscosities.

### 3.1. Septum Characterization

The results (Figure 6B) indicated that the samples without the compression rings could not withstand one puncture with the 30 Ga needle and leaked at a low backpressure of 5 kPa. In contrast, the samples with the compression rings withstood up to ~65,000 punctures without leakage at 100 kPa backpressure using the 30 Ga needle. However, using the 27 Ga needle reduced the number of punctures without leakage against the same backpressure to 55, sufficient for many therapeutic developments and clinical applications (e.g., [47,48]). A comparison between the results of the 30-Ga-needle puncture tests with and without the compression rings indicates its significance in increasing the lifetime of the septa. All septum puncture experiments were performed on 1-mm thick septa. Testing on 0.5-mm-thick septa showed poor functionality at low backpressures, even with the aid of compression rings. This could be due to fabrication limitations in controlling the thickness of the compression ring and the buckling of the septum due to the lateral force of the compression.

To explore the deformation of the septum due to imposing 100 kPa pressure a 2D-axisymmetric finite element analysis was performed using COMSOL multiphysics^®^. The septum model was fixed at the perimeter from top and bottom, simulating the septum stopper and compression ring, and matching the aforementioned dimensions. A boundary load of 100 kPa was applied to the bottom surface. The results showed a deformation of 111 µm for the septum center due to the backpressure, demonstrating insignificant effects of pressure on septum deformation and functionality.

### 3.2. Storing Membrane Characterization

The results indicated that the capacities of the microreservoirs were 1.15 ± 0.12, 9.63 ± 0.12, and 100.04 ± 9.43 µL (mean ± SD) for target volumes of 1, 10, and 100 µL, as shown in Figure 6C. Figure 6D shows the average of the extracted volume during pumping and resting for the three nominal capacities, normalized by the total capacity of each. Each data point is an average of the results from three devices with three replicates (N = 9). The results showed that for all steps, the forward flow due to membrane force was zero, and the average backward flow was not significant; 2.02% of the overall capacities. The backward flow at each step occurs relatively quickly after releasing pneumatic pressure (<2 min for all cases), ensuring the stable behavior of the storing membrane for long-term applications. Further, no significant difference in the backward flow was observed across different extraction percentages, except for the last step, which defines the total extraction percentage of the microreservoirs. The average total extraction percentage was 94.83% of the overall capacity.

Samples were also created with membranes twice the thickness of the original for each capacity (N = 9). Experiments showed significantly more average backward flows; 3.8% of the overall capacities. This significant difference (*p* < 0.001) is directly related to the thickness of the membranes. As the membrane becomes thicker, its resistance against deformation increases, resulting in a higher restoring force and a greater backward flow when the pneumatic pressure is released. However, we hypothesize that the total extraction percentage is a function of the discrepancy between the surface area of the storing membrane and the substrate under it. If the membrane surface area is larger than the substrate, when fully pushed down by the pneumatic pressure, the membrane wrinkles on the substrate and resists against further applied pressure. When the pneumatic pressure is released, these areas induce a restoring force and, consequently, a backward flow. On the other hand, if the membrane surface area is smaller than the substrate, there is space between the membrane and the substrate that cannot be swept by the membrane. However, this space can be swept by further pressure, which restores when the pressure is released and causes the backward flow.

The results of the discharge/refill experiment showed that the storing membranes could withstand at least 2000 cycles (N = 4). No further cycles of discharge/refill experiments were conducted, because 2000 cycles exceed the typical lifetime of micro-scale reservoir-based drug delivery devices; with daily refills, the reservoir could run for more than five years.

### 3.3. In Vitro Characterization

The results of *in vitro* experiments to replicate implantable application (Figure 6E) with dyed DI water as the working fluid (µ = 1.1 cP) showed an average backflow of 1.69% and overall extraction percentage of 95.53%. Comparing these results and those of the membrane characterization shown in Figure 6D indicates that using the microreservoirs in implant environments does not significantly impact the performance (0.33% difference of the total capacity). Furthermore, using model drugs with viscosities of 19.8 and 151.7 cP, the average backflows were 1.98% and 2.59%, with overall extraction percentages of 95.85% and 95.40%, respectively. The results showed that the microreservoirs can operate independent of the drug viscosity in a simulated subcutaneous implant environment.

The results of *in vitro* experiments to simulate transdermal application (Figure 6F) with dyed DI water as the working fluid revealed an average backflow of 2.06% and an overall extraction percentage of 96.11%. Comparing these results and those of the membrane characterization shown in Figure 6D indicates that using the microreservoirs in transdermal environments does not significantly impact the performance (0.04% difference of the total capacity). Further, using model drugs with viscosities of 19.8 and 151.7 cP, the average backflows were 2.24% and 2.33% with overall extraction percentages of 97.36% and 96.64%, respectively. The results indicated that the microreservoirs operate in a simulated transdermal drug delivery environment, independent of the working fluid viscosity.

## 4. Discussion

Drug delivery control is crucial to ensure that administered drugs are delivered sustainably with the desired dosage and therapeutic efficacy with minimal side effects. Micro-scale reservoir-based drug delivery devices have been used in implantable and transdermal systems. These systems usually include a microreservoir for drug storage, a micropump for fluidic actuation, and a microchannel to deliver the drug to the target organ (e.g., hollow microneedles, catheter microtubing). A modular microreservoir provides an easy-to-use platform for the drug storage component to be readily integrated into different pumping mechanisms.

The miniature, thin, scalable, and modular microreservoirs presented here were designed for applications ranging from mice to humans with capacities of 1, 10, and 100 µL, which can be readily fabricated to specific sizes in between or scaled up to larger sizes with scaling of membrane thickness following Equation (1). These microreservoirs can serve for both implantable and transdermal applications. It has been reported that an implantable prototype should weigh <10% of the animal’s body weight [49], which does not exceed 35 g for an adult mouse as the smallest laboratory animal [50]. Our largest microreservoir with 100 µL capacity weights only ~0.5 g (including the stored fluid), which leaves ~3 g for other components of the implantable micro-scale device to be integrated (e.g., micropumps). Similarly, the surface area of a subcutaneously implanted object should involve <10% of the animal’s surface area for a surgery to be a minor procedure [51]. Since the mouse surface area is 7000 mm^2^ [52], our largest microreservoir with 100 µL capacity takes only 2.4% of the animal’s surface area (167 mm^2^), leaving 533 mm^2^ for other features to be integrated. The above calculations are for implantable devices, while transdermal devices do not need to meet such stringent standards. Further, all the microreservoirs are only 3-mm thick, thin enough for both implantable and transdermal applications.

We presented a novel fabrication technique that combines SLA 3D-printing of a biocompatible resin and parylene-C deposition on sacrificial layers; the latter has been used in the literature to create parylene cavities [28]. Features as small as 350 × 250 µm^2^ can be fabricated using SLA 3D-printing with a biocompatible resin [53] by simply uploading the Computer-aided design (CAD) file to the printer. This fabrication technique significantly facilitates the integration of the presented microreservoir with pumping mechanisms and other features in the system through simple modifications in the configuration in CAD file design. Further, since SLA-printed parts can be scaled by minimal changes in the CAD file, and parylene-C deposition is a conformal process, this fabrication process enables scalability with a minimum effort. The SLA 3D-printing technique also allows the substrate to be curved to comfortably come into contact with the skin for transdermal applications. Using standard fabrication techniques such as SLA, parylene-C deposition, micro-molding, and the adhesive bonding of components enables low-cost manufacturing.

The microreservoir can be integrated into pumping or suction mechanisms for various applications in implantable and transdermal drug delivery or sampling. The fluid in the storing area can be actuated either directly from the top of the microreservoir membrane or with the outlet microtubing. Both these pumping mechanisms were used in this report to find different forward and backward flows due to membrane forces (see Figure 4 and Figure 5). Other actuation mechanisms can be utilized for pumping by direct pressure on the storing membrane, such as thermal [54], electrolysis [55], phase change [8], or manual [25]. Further, all the aforementioned types of micropumps can be integrated into the outlet microtubing as their inlet port (e.g., [56]). Micropumps can also be fabricated directly around the outlet microtubing at the outlet to minimize leakage due to microfluidic interconnections (e.g., [57,58], iPRECIO^®^ SMP-300), which has been reported to be challenging [59].

For implantable applications of the current design, the outlet microtubing can be up to 500 µm OD (since the storing substrate is 700 µm tall), or it can be coupled to different tubing sizes, depending on the applications. However, minimal changes in the CAD design enables using larger tubings with no coupling components. For transdermal applications, the outlet microtubing can be eliminated and replaced by a platform with an array of hollow microneedles at the bottom of the storing area. An example of such a platform is presented in [60], where an array of microneedles was fabricated with micro-electromechanical system technology attached to a PDMS structure, and optimized for integration to storing and pumping mechanisms.

Although the prototypes presented here are microreservoir modules with a storing area and a refill port for drug delivery, they can be decoupled for different applications. The pre-compressed ultra-thin septum concept capable of withstanding thousands of leakage-free injections can be used for various devices with a variety of applications, including drug delivery, lab-on-a-chip (LOC), point-of-care (POC) diagnosis, etc. Furthermore, for applications with a passive release, the microreservoir can be implemented without requiring any pumping mechanism’s presence, where the diffusion occurs through microtubings or hollow microneedle outlets [7,61]. These microreservoirs can also be used for sampling by the integration of suction mechanisms, especially for transdermal applications. Finally, the microreservoirs can be readily used in LOC or POC diagnostic systems using any of the aforementioned pumping mechanisms.

## 5. Summary and Conclusions

We developed a stand-alone refillable, scalable microreservoir designed for integration into pumping mechanisms, optimized for implantable, and transdermal drug delivery. The microreservoir structure has two main components: the refill port for refilling injections through a septum, and the storing area for long-term drug storage. The microreservoir was built upon an SLA 3D-printed biocompatible structure, which enables miniaturization and scalability of the design, supporting applications in small laboratory animals and translational opportunities. The storing membrane was fabricated with parylene-C using PEG as the sacrificial layer, over the parylene-C-coated substrate. The silicone septum thickness was reduced to 1 mm with excellent durability by adding a compression ring to the septum cap to induce internal compression within the septum. Experimental characterization results demonstrated no leakage for the 1 mm-thick septum after up to ~65,000 injections with a 30 Ga needle at up to 100 kPa backpressure, which is four times greater than the maximum backpressure in humans thereby offering a useable lifespan of more than 5 years. The scalability of the design and fabrication process was demonstrated by the fabrication of three different microreservoir capacities (1, 10, and 100 µL) while keeping the overall thickness of 3 mm. The experimental characterization results showed that the membrane causes zero forward flow and an average backward flow of as low as 2% of the total capacity. This feature enables precise control over flow rates when the microreservoir is integrated into pumping mechanisms. Future work will integrate this microreservoir platform to enable novel POC diagnostics, LOC, and drug delivery applications. Furthermore, *in vitro* experiments demonstrate the suitability of the device for both subcutaneous implantable and transdermal drug delivery for a broad range of drug viscosities.

## Supplementary Materials

The following are available online at https://www.mdpi.com/2072-666X/11/7/648/s1, Figure S1: Detailed design and dimension of microreservoir components.
