# Supplementary material for: A 3D-Printed Modular Microreservoir for Drug Delivery
Source: Micromachines (Basel). 2020 Jun 30;11(7):648. doi: 10.3390/mi11070648 (PMC7407798; doi:10.3390/mi11070648)
Supplement: Supplementary file 1 [file micromachines-11-00648-s001.pdf]

Supplementary Material

A 3D-Printed Modular Microreservoir for Drug Delivery

Farzad Forouzandeh, Nuzhet N. Ahamed, Meng-Chun Hsu, Joseph P. Walton, Robert D. Frisina and David A. Borkholder

Detailed design and dimension of microreservoir components

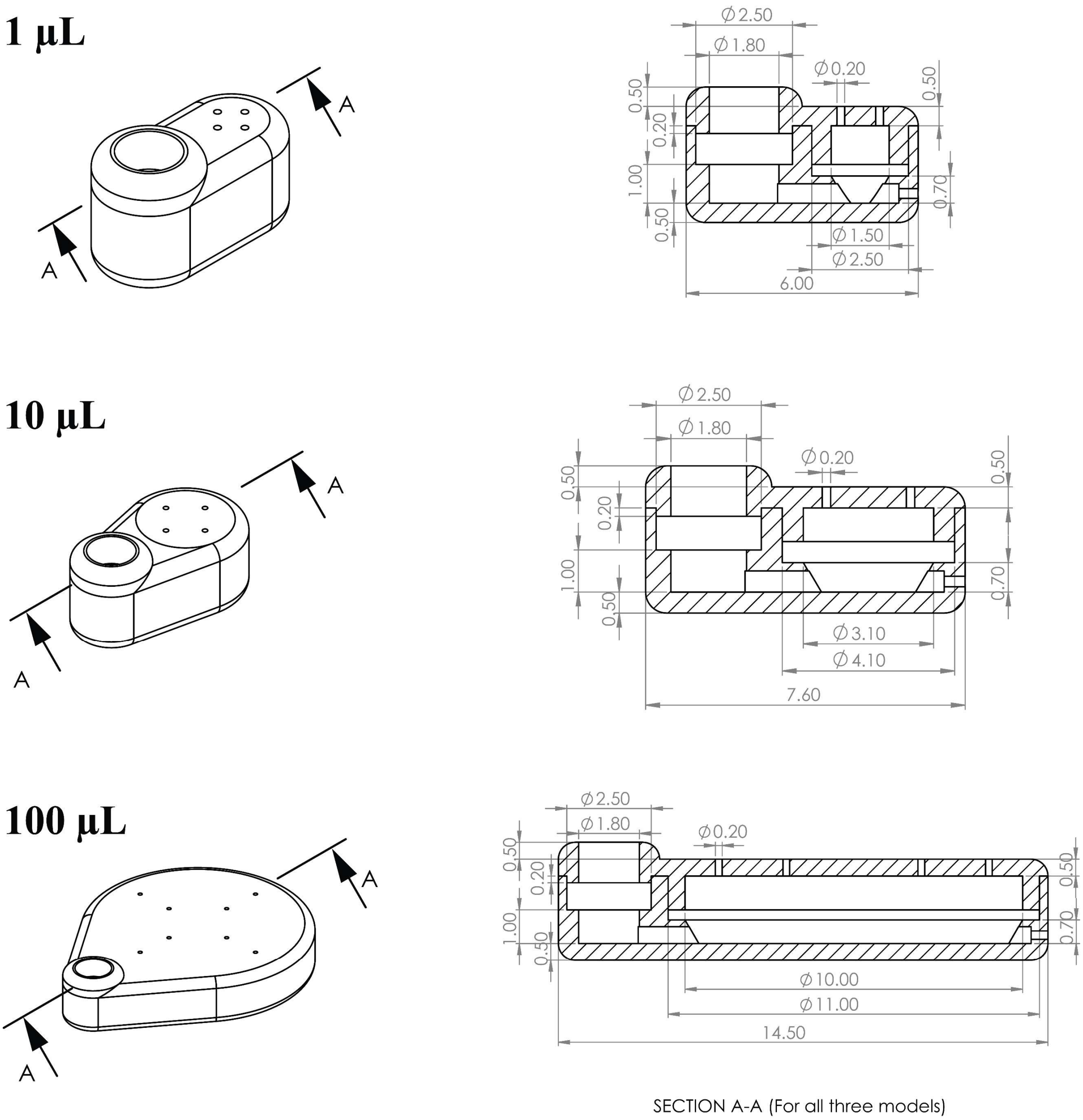

Figure S1. Iso view of three different stand-alone microreservoirs along with cut view at section A-A. Dimensions all in mm. Dimensions not to be scaled.
